# Robotic liver surgery: enhancing immune competence and minimizing postsurgical inflammation

**DOI:** 10.1007/s00464-025-12195-1

**Published:** 2025-09-26

**Authors:** Julia Nagelschmitz, Thomas Wartmann, Severin Gylstorff, Ahmed Sanin, Ronny Otto, Jörg Arend, Mareike Franz, Mirhasan Rahimli, Andrew A. Gumbs, Ulf D. Kahlert, Frederike Stelter, Roland S. Croner

**Affiliations:** 1 Medical Faculty and University Medical Center Magdeburg, Clinic for General-, Visceral-, Vascular-, and Transplantation Surgery, Leipziger Str. 44, 39120 Magdeburg, Germany; 2 Experimental Radiology, University Clinic for Radiology and Nuclear Medicine, Medical Faculty and University Medical Center Magdeburg, Leipziger Str. 44, 39120 Magdeburg, Germany; 3 Institute for Quality Assurance in Operative Medicine GmbH, Leipziger Str. 44, 39120 Magdeburg, Germany; 4 Service de Chirurgie Digestive Minimale Invasive, Hôpital Antoine Béclère, Assistance Publique-Hôpitaux de Paris, 92140 Clamart, France; 5 Research Campus STIMULATE, Otto-Von-Guericke University Magdeburg, Otto-Hahn-Straße 2, 39106 Magdeburg, Germany

**Keywords:** Robotic liver surgery, Inflammation, Immune competence

## Abstract

**Background:**

In recent years, more complex robotic-assisted liver resections (RLR) have been performed, providing a viable alternative to open liver resection (OLR). While the short-term benefits of minimally invasive surgery are well known, including reduced blood loss and shorter hospital stay, the inflammatory response to different surgical approaches remains poorly understood.

**Methods:**

This study examines the immune response in peripheral blood and local liver and peritoneal tissue during and after liver surgery in 22 patients (11 in each group). The study analyzes clinical and laboratory parameters, leukocyte activation, and cytokine/chemokine levels before and after liver parenchyma dissection using L-selectin shedding assay and FACS multiplex analysis panel.

**Results:**

In the perioperative course, systemic and local liver cytokine levels of IL-6 and IL-10 are reduced in RLR. The laparotomy itself resulted in higher baseline levels of IL-6, IL-8, CXCL10, IFNγ, TGFβ1, and IL-1β in local liver tissue of the OLR group. After liver parenchyma dissection, RLR patients exhibited reduced levels of IL-6, IL-8, IFNγ, MCP1, IL-1β, TGFβ1, and CXCL10 in the liver compared to the OLR group. In the late postoperative course from postoperative day (POD) 5–20, systemic chemokine MCP1 was reduced, alongside a decrease of CD4^+^/CD8^+^ lymphocytes and higher L-selectin shedding capacity in the RLR group from POD5 onwards.

**Conclusion:**

These findings suggest that RLR preserves immune competence more effectively than OLR in the peri- and late postoperative course. The reduced systemic and local inflammatory response may be the result of less tissue damage with reduced cytokine release, highlighting the value of less traumatic surgery applied by robotic systems during clinical practice.

**Graphical Abstract:**

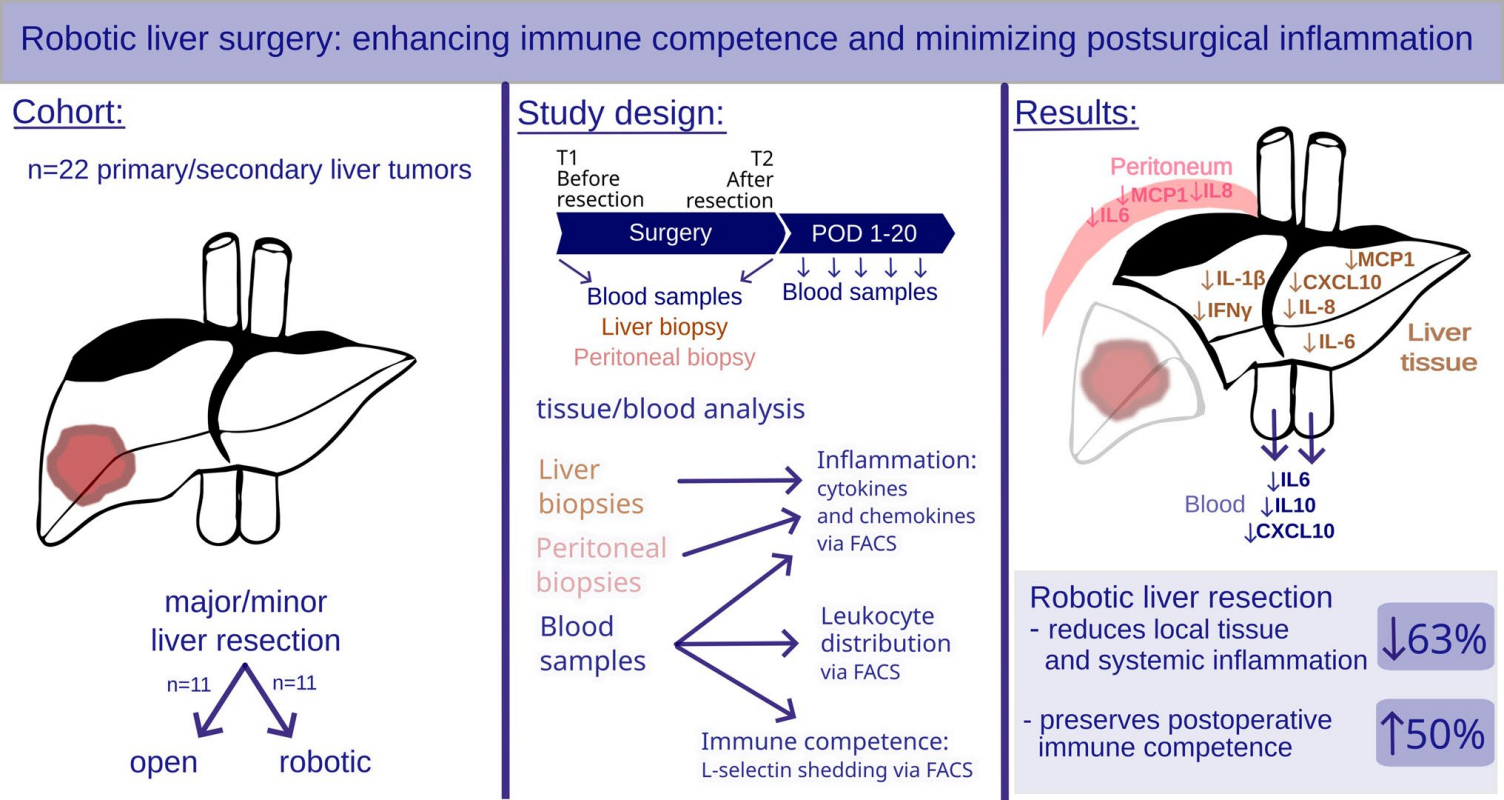

**Supplementary Information:**

The online version contains supplementary material available at 10.1007/s00464-025-12195-1.

Minimally invasive liver surgery (MILS) has gained increasing attention due to its favorable short-term patient outcome. Laparoscopic and robotic minor or major liver resections are associated with reduced blood loss, less postoperative major morbidity, improved quality of life, and shorter overall hospital stay [1–4]. Comparison between minimally invasive and open surgical approaches has demonstrated similar, and in some cases, slightly improved long-term outcomes regarding mortality, although these findings are not always statistically significant [5–7]. Another potential advantage of minimally invasive liver resection is the ability to initiate adjuvant chemotherapy earlier (42 vs 63 days, respectively), likely due to a shorter recovery time [2, 8]. Patients with impaired liver functions, such as liver cirrhosis, particularly benefit from minimally invasive surgical resections [7]. The choice between robotic or laparoscopic approaches has only marginal influence on perioperative and long-term patient outcomes [9, 10]. However, robotic-assisted surgery offers distinct advantages in performing complex surgical procedures, such as biliary reconstruction in patients with cholangiocellular adenocarcinoma (CCA) or vascular reconstructions [11].

Despite the well-documented benefits of MILS, less is known about its impact on the inflammatory response compared to open liver resections (OLR). Surgical trauma induces a hyperinflammatory response, characterized by the release of cytokines, including IL-6, IL-8, and IL-10 [12]. Excessive cytokine release can trigger a systematic inflammatory response syndrome, an acute proinflammatory state, while a subsequent compensatory anti-inflammatory response leads to postoperative immunosuppression [13]. Cytokine levels typically peak on the first postoperative day due to prolonged surgical duration, excessive tissue trauma, and high blood loss. This exaggerated systemic inflammatory response is associated with postoperative liver injury, organ failure, and increased hospital length of stay [14, 15].

The first study assessing the differences in inflammatory responses between MILS and OLR was conducted in a porcine model in 2002. It demonstrated significantly higher levels of IL-6 and TNFα in the OLR group [16]. Subsequent human studies further supported this trend: in early-stage hepatocellular carcinoma (HCC) resections, laparoscopic surgery was associated with lower GM-CSF, IL-6, IL-8, and MCP1 levels compared to open liver surgery [14]. Similarly, in colorectal liver metastasis (CRLM) resections, laparoscopic surgery resulted in reduced systemic inflammation as evidenced by lower levels of HMGB-1, cfDNA, IL-6, CRP, and MIP-1β [17]. Comparable findings have been reported in gastrointestinal surgeries. For instance, Yu et al. (2010) demonstrated higher levels of IL-1ß in the open gastrectomy group compared to the laparoscopic group [18]. Furthermore, Zhu et al. (2019) also reported lower CRP and TNFα levels in serum and peritoneal tissue after laparoscopic versus open colon carcinoma resection [19]. Additionally, robotic colorectal surgery was associated with significantly reduced inflammatory markers such as IL-6 and procalcitonin up to postoperative day (POD) 3 [20].

There is growing evidence suggesting a link between heightened systemic inflammation and poor oncological outcomes. Elevated IL-6 levels on POD1 are considered a risk factor for HCC recurrence [14]. High IL-1β and TNFα levels have also been implicated in an increased risk of local gastric cancer recurrence, particularly in open resections [18].

In this study, we provide the first comparative analysis of systemic and local inflammatory responses in open and robotic minor and major liver resections for different tumor entities. We assessed leukocyte populations and their activation, as well as local and systemic cytokine production, in the peri- and postoperative period. This study will pave the way toward a better understanding of inflammatory response to surgical trauma of different surgical approaches. The preserved immunocompetence of patients undergoing minimally invasive liver surgery will further strengthen its role as a safe and favorable approach for liver tumor resection.

## Methods

### Study population

Beginning in October of 2023, we recruited patients undergoing scheduled open or minimally invasive liver surgery at our hospital after obtaining informed consent. The exclusion criteria comprised the following: age under 18 years, no contractual capacity, language barrier, pregnancy, immunosuppressive medication, prior organ transplantation, emergency surgery, and simultaneous multi-visceral resection. All patients followed the same preoperative procedure according to the clinical standard. Patient data were obtained from our medical information system. The decision between OLR and RLR was based on individual patient criteria. Minor resections involved < 3 segments, while major resections involved ≥ 3 liver segments, classified by the Brisbane terminology [21]. After surgery, the rate of postoperative complications was assessed according to the Clavien–Dindo classification.

### Sampling of tissue, blood and drainage fluid, leukocyte isolation from blood

**Fig. 1:**
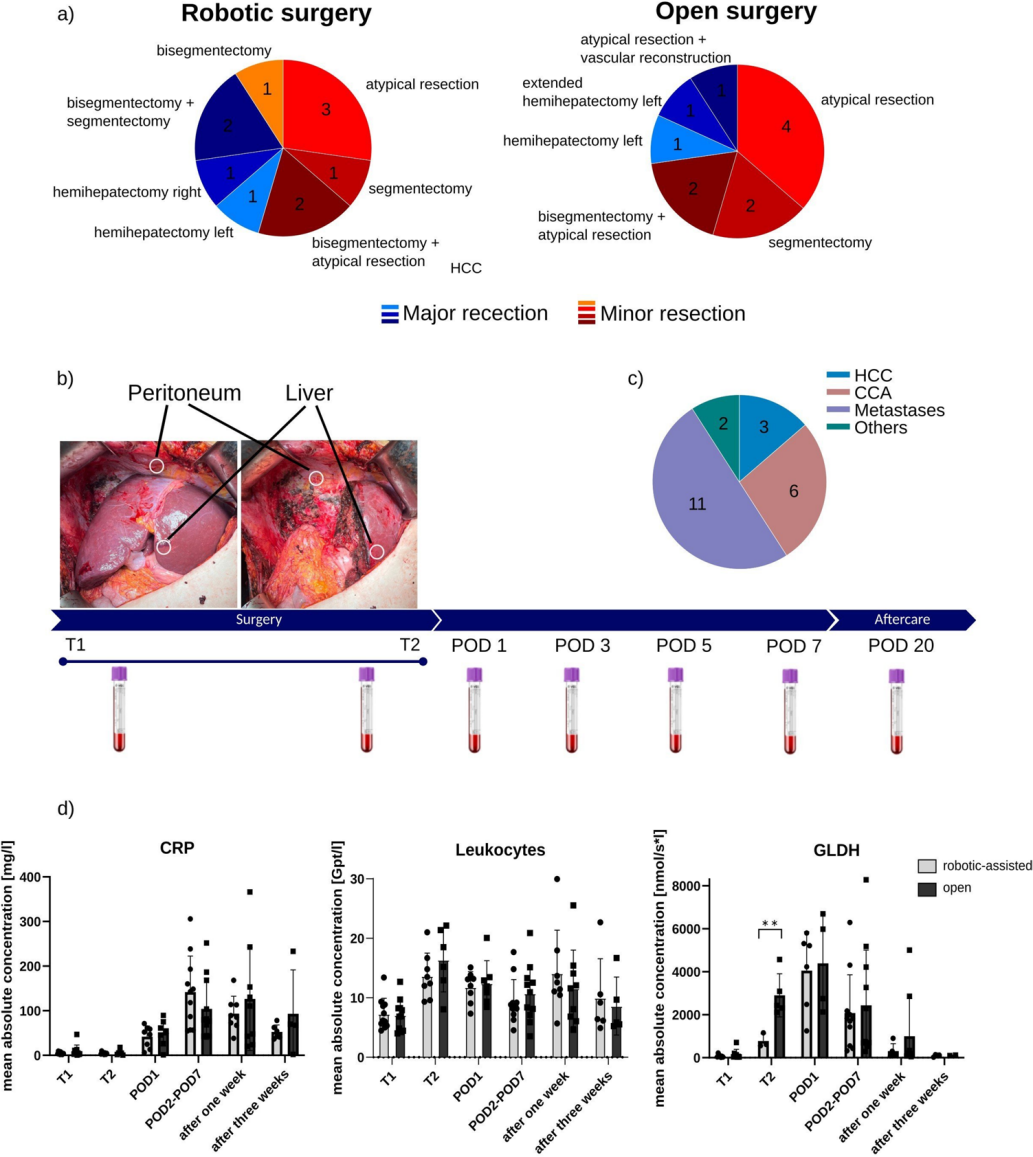
Baseline patient characteristics and laboratory parameters Panel (**a**) pie chart of the extent of the liver resection in open and robotic liver surgery divided into major (blue) and minor (red) resection; (**b**) timeline of sample acquisition; (**c**) shows a pie chart of the overall tumor entities indicating for a liver resection; (**d**) mean absolute concentration of CRP, leukocyte count and GLDH measured in the peri- and postoperative course

The samples of liver and peritoneal tissue were directly retrieved from surgery in the initial phase as soon as the organ was accessible (T1) and at the end of surgery (T2). The liver sample was obtained from healthy non-tumorous tissue near the operation site, approximately in a 5 cm surrounding in a good accessible area. The peritoneal sample was obtained from peritoneal parts adherent to the liver or the diaphragm in the right upper abdomen (Fig. 1b). Tissue samples were minced and stored before further use at − 80 °C.

Blood was collected at T1 and T2 and POD 1, 3, 5, 7, and 20 in an EDTA tube and in a tube without additives (Fig. 1c). After clotting for 45 min, the serum was centrifuged at 1500xg for 15 min and stored at − 80 °C for later use. The EDTA blood was treated with erythrocyte lysis buffer, incubated for 10 min, and centrifuged at 380xg for 10 min. The pellet was resuspended, and the above-mentioned steps were repeated until the pellet was completely white. The pellet was once washed with PBS, centrifuged at 380×g for 10 min, resuspended in freezing medium (FCS with 10% of DMSO) and stored at − 80 °C.

### Staining for leukocyte subpopulations

Cryopreserved isolated leukocytes were thawed in a water bath at 37 °C. For Fluorescence Activated Cell Sorting (FACS) analysis, leukocytes were divided into unstained, lymphocyte panel, monocyte panel, and leukocyte stimulation (see ‘Stimulation of leukocytes’). Antibodies and dilutions were described in detail in Table S1.

**Fig. 2:**
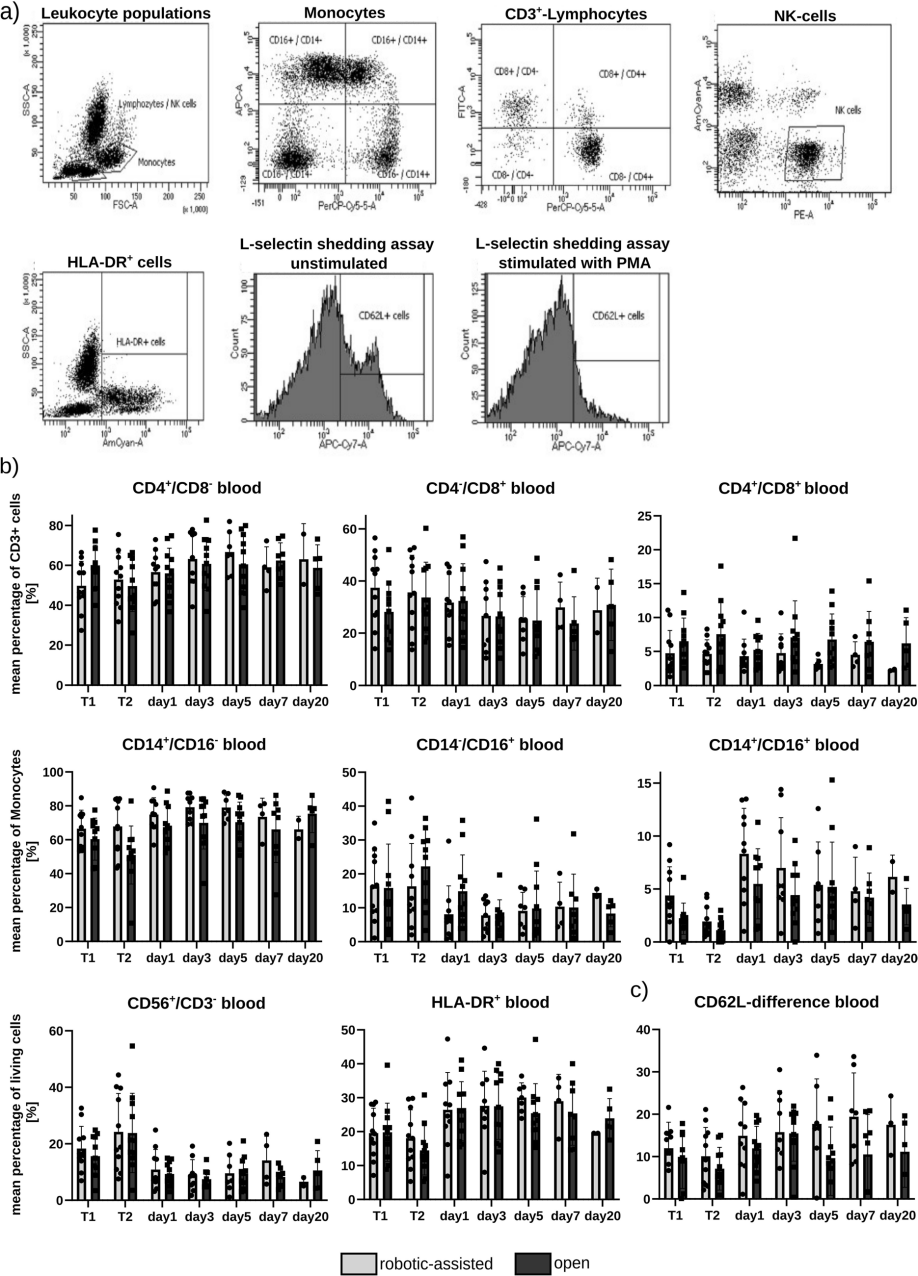
Gating and results of the leukocyte population and inflammatory leukocyte stimulation assay are depicted. (**a**) Gating of the leukocyte subpopulations for flow cytometry; (**b**) fraction of certain leukocyte subpopulations, namely CD3^+^-Lymphocytes (CD4^−^/CD8^+^, CD4^+^/CD8^−^, CD4^+^/CD8^+^), Monocytes (CD14^−^/CD16^+^, CD14^+^/CD16^−^, CD14^+^/CD16^+^, HLA-DR^+^) and NK cells (CD56^+^/CD3^−^), measured in blood on T1, T2, POD1, POD3, POD5, and POD20; (c) results of the leukocyte activation at the beginning of surgery (T1), end of surgery (T2) and the postoperative course displayed as the difference of CD62L-positive cells in the unstimulated and stimulated sample

The samples were washed in 750 µl PBS (350xg, 5 min, 4 °C). Staining tubes were then resuspended in 100 µl of a live/dead staining (Zombie Violet) and incubated for 30 min at room temperature (RT). The staining tubes were washed with FACS Buffer, resuspended in 100 µl FACS Buffer (PBS, 2% FCS, 2 mM EDTA) and mixed with 8.5 µl of antibody mastermix I for lymphocyte panel and 7 µl of antibody mastermix II for monocyte panel (see Table S1), respectively. After 30 min of incubation at RT, stained tubes were washed twice with FACS Buffer and analyzed by flow cytometry on a FACSCanto II (BD Biosciences). 10.000 events gated for the living cells were measured for each sample (Fig. 2a).

### Stimulation of leukocytes

To evaluate the immune competence of patients during and after the surgery, leukocyte stimulation assay was conducted using the leukocyte activation marker CD62L (L-selectin). This marker, among other selectins, is responsible for the migration of leukocytes, especially the lymphocyte homing, to the site of inflammation [22]. During this process, CD62L is shed from the cells, which is why the presence of CD62L on leukocytes can be measured and interpreted as a marker for the activation of immune cells. The effect of inflammation can be simulated using a stimulation agent like PMA, resulting in the shedding of CD62L. Thawed isolated leukocytes were washed and resuspended in 175 µl of Phorbol-12-myristat-13-acetat (PMA, 75 ng/ml in PBS) and incubated for 3 h at RT in the dark. Afterward, the cells were washed with FACS Buffer and centrifuged at 350xg for 5 min. The stimulated leukocytes were then treated according to the established staining protocol, beginning with the live/dead staining. In the next step, the cells were only incubated with the CD62L: APC/Cy7 antibody (see Table S1) and measured by flow cytometry for both arranged panels.

### Homogenization of tissue samples

Liver and peritoneal tissue samples (approximately 50 mg each) were weighed and mechanically disrupted after thawing. For liver tissue, Lysing Matrix D was used; for peritoneal tissue, Lysing Matrix E (MP Biomedicals, 1,169,140-CF) was used. In every digestion tube, additionally, Cell Lysis Buffer (Cell Signaling, #9803) mixed with 1 mM PMSF in the ratio 1:10 was added.

The tissue was homogenized using a Fastprep-24 5 G device (MP Biomedicals, 3 cycles à 30 s, 6 m/s), then centrifuged at 1000×g for 30 s. The supernatant underwent ultrasonic homogenization for 30 s, followed by centrifugation at 14.000×g for 10 min. The supernatant was stored at − 80 °C.

### Multiplex assay panel

A multiplex assay panel was conducted on tissue homogenates and patient up to POD5 using the LEGENDplex HU Essential Immune Response Panel (13-plex) with a v-bottom plate for flow cytometry. The samples were prepared and the measurement carried out according to the manufacturer’s protocol (See supplementary data). The plate was read on a BD FACSCelesta (BD Biosciences) and analyte concentrations were evaluated using the LEGENDplex Data Analysis Software Suite (Biolegend).

### Statistical analysis

The statistical analysis was performed using SPSS by means of a Student t-test (with Welch’s correction). *p* < 0.05 was taken as significant. Because of the small sample size and elevated risk of type II error, we further calculated the effect size using Hedge’s g to consider the non-symmetric sample size. A moderate effect was defined as ≤ − 0.5/ ≥ 0.5, and a strong effect was defined as ≤ 0.8/ ≥ 0.8. Data are provided in Table S3 for the relevant parameters.

## Results

### Patient cohort and clinical parameter

In this prospective, non-randomized study, 22 patients who underwent an open or robotic-assisted liver resection at university hospital in Magdeburg have been recruited. The cohort consists of 14 male and 8 female patients with an equal distribution of age (62 ± 9.70 OLR vs. 64.6 ± 10.3 years RLR) and BMI (31.1 ± 5.72 OLR vs. 27.6 ± 4.68 kg/m^2^ RLR) among the groups. The ASA classification was equally distributed between both groups (ASA2: 5 OLR vs 4 RLR; ASA3: 6 OLR vs. 7 RLR). Most of the patients had a malignant tumor, except for one patient with a giant biliary adenofibroma in the liver, who underwent a pericystectomy with vascular reconstruction of the middle and left hepatic vein. Of the 21 patients with malignant tumors, 9 had primary liver tumors (3 HCC, 6 CCA), 11 had liver metastases mostly from colorectal origin, and one patient had a gallbladder carcinoma (Fig. 1a). In our study cohort, 12 patients (54.5%) had already undergone major abdominal surgery (6 patients each group), mainly those with a CRLM who had previously undergone resection of the primary colon cancer (Fig. 1c, Table 1). Five of these patients (22.7%) had previously undergone liver surgery for a malignant primary or secondary liver tumor, all within the open liver resection group. There was no difference in the quality of surgical resection between RLR and OLR groups. All malignant tumors were resected with microscopic tumor-negative margins (R0). In the RLR group, there were significantly more patients with neoadjuvant chemo- or radiotherapy, because more liver metastases were resected with the robotic approach (4 OLR vs. 7 RLR).

**Table 1:** Patient cohort, clinical parameter, and laboratory parameters

	OLR	RLR	All	*p*	*n*
Sex				0.659	22
Male	6 (54.5)	8 (72.7)	14 (63.6)		
Female	5 (45.5)	3 (27.3)	8 (36.4)		
Age at time of surgery [years]	62.4±9.70	64.6±10.3	63.5±9.83	0.600	22
BMI [kg/m^2^]	31.1±5.72	27.6±4.68	29.3±5.42	0.126	22
ASA-score				1.000	22
2	5 (45.5)	4 (36.4)	9 (40.9)		
3	6 (54.5)	7 (63.6)	13 (59.1)		
Previous surgery					
Previous surgery of the abdomen	6 (54.5)	6 (54.5)	12 (54.5)	1.000	22
Previous surgery of the liver	5 (45.5)	0 (0.00)	5 (22.7)	0.035**	22
Neoadjuvant chemo-/radiotherapy	2 (18.2)	8 (72.7)	10 (45.5)	0.032**	22
Duration of surgery [min]	221±69.4	348±47.8	285±87.1	<0.001**	22
Blood loss [ml]	687±708	345±283	516±554	0.161	22
Time of Pringle maneuver [min]	33.0	52.0±18.2	47.2±17.6		4
Clavien-Dindo-Score				0.797	19
0	3 (27.3)	4 (36.4)	7 (31.8)		
1	3 (27.3)	1 (9.09)	4 (18.2)		
2	0 (0.00)	1 (9.09)	1 (4.55)		
3	4 (36.4)	3 (27.3)	7 (31.8)		
Duration of stay in intensive care unit	11.3±26.8	6.25±8.05	8.60±18.6	0.647	15
Catecholamine therapy after surgery	2 (18.2%)	4 (36.4%)	6 (27.3%)		6
Mechanical ventilation after surgery	2 (18.2%)	3 (27.3%)	5 (22.7%)		5
Duration of hospital stay [days]	20.1±20.1	16.1±9.97	18.1±15.6	0.563	22
Rehospitalisation in the first 30 days	2 (18.2)	2 (18.2)	4 (18.2)	1.000	22

Seven patients received atypical resection (3 OLR vs. 4 RLR), 3 patients received segmentectomy (1 OLR vs. 2 RLR), 1 patient received bisegmentectomy in the OLR group, 4 patients received bisegmentectomy with additional atypical resection (2 OLR vs. 2 RLR), 2 patients received left hemihepatectomy (1 OLR vs. 1 RLR), and one patient received a right-sided hemihepatectomy in RLR and another patient received an extended left-sided hemihepatectomy in the OLR group (Fig. 1a). Seven patients underwent major liver resection (3 OLR vs. 4 RLR) and 15 patients received minor liver resection (8 OLR vs. 7 RLR; see Fig. 1a). Robotic surgery took significantly longer compared to the OLR group (221 ± 69.4 min OLR vs 348 ± 47.8 min RLR, *p* ≤ 0.001, *g =* − 2.13), with a slightly higher rate of postsurgical mechanical ventilation (2 vs. 3 patients) and catecholamine support (2 vs. 4 patients). However, RLR group patients had shorter treatment in the intensive care unit (11.3 ± 26.8 days in OLR and 6.25 ± 8.05 days in RLR) and a shorter overall hospital stay (20.1 ± 20.1 OLR vs. 16.1 ± 9.97 RLR,). Intraoperative blood loss was reduced in RLR (345 ± 283 vs. 687 ± 708, *p =* 0.161, *g =* 0.05), whereas postsurgical complications and rehospitalization were similar between both groups (Table 1). Laboratory parameters were analyzed in both groups displaying no difference in the level of leucocyte and CRP values in the postoperative course (Fig. 1d). Liver parameters as GLDH were higher in the OLR group at T2 (Fig. 1d; 2903 ± 1002 nmol/s OLR vs. 779 ± 331 nmol/s RLR, *p =* 0.007**, *g =* 2.53). Further laboratory parameters such as bilirubin, INR, and transaminases show no significant difference and are displayed in Fig. S1 in the supplementary dataset.

### Leukocyte population characterization and stimulation efficacy

The systemic inflammatory response was initially analyzed based on the following cell populations: lymphocytes (CD4^−^/CD8^+^, CD4^+^/CD8^−^, CD4^+^/CD8^+^), monocytes (CD14^−^/CD16^+^, CD14^+^/CD16^−^, CD14^+^/CD16^+^) and NK cells (CD56^+^/CD3^−^). These populations were analyzed in peripheral blood on T1, T2, POD1, POD3, POD5, and POD20. CD4^+^/CD8^+^ lymphocytes display reduced levels in the robotic group in all measured time points, with intensified reduction with a strong effect size in the late postoperative course from POD5 (*g =* 1.17) to POD20 (*g =* 0.84). Levels of differentiated CD4^−^/CD8^+^ or CD4^+^/CD8^−^ were similar between OLR and RLR groups. Monocytes and NK cells displayed similar population distributions in both groups.

Additionally, leucocyte activation via CD62L (L-selectin) shedding was analyzed. Stimulated samples with PMA indicate the activability of the leukocytes and the immune competence of the patients at different points of time (Fig. 2c). While being almost similar at the beginning of surgery (8.91 ± 6.42 vs 12.0 ± 6.12, *p =* 0.264, *g =* − 0.49), the tendency for a higher CD62L difference (unstimulated to stimulated) in patients undergoing RLR is getting more prominent in the first postoperative days and maintains this tendency with a strong effect size for a better immune competence from POD 5 (*g =* − 0.96) until POD 20 (*g =* 0.8).

### Cytokine and chemokine expression in the peripheral blood

**Fig. 3:**
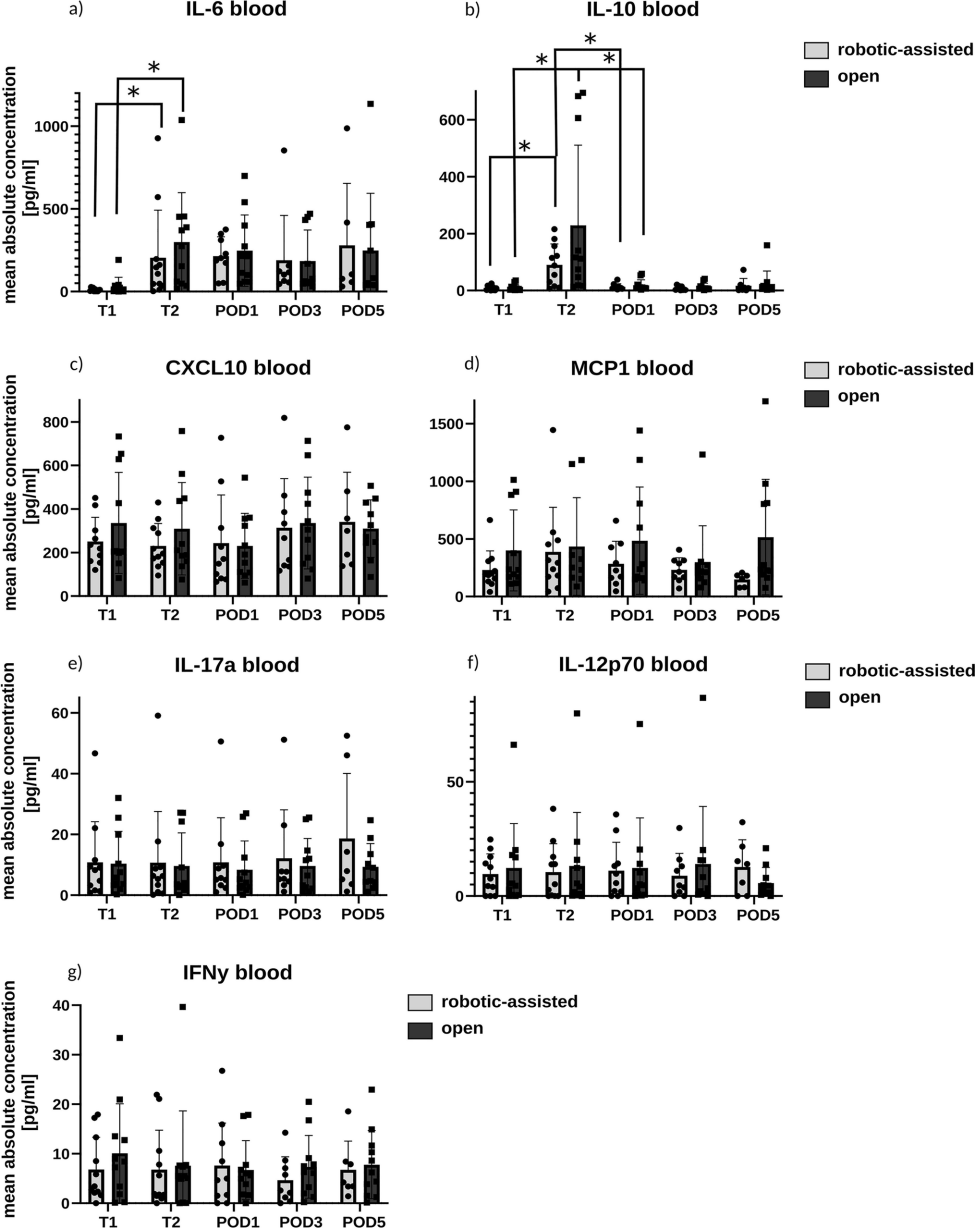
Concentration of selected regulatory proteins in the peripheral blood on the initial phase of the surgery (T1), at the end of surgery (T2) as well as on the postoperative days (POD) 1, 3 and 5. (**a**) The mean concentration of IL-6; (**b**) the mean concentration of IL-10; (**c**) the mean concentration of CXCL10; (**d**) the mean concentration of MCP1; (**e**) the mean concentration of IL-17a; (**f**) the mean concentration of IL-12p70; (**g**) the mean concentration of IFNy. For an enhanced visual representation of our data, outliers, defined as extreme values differentiating fivefold of the interquartile range from the lower or upper quartile, have been excluded from the figures

Furthermore, the concentration of different cytokines and chemokines measured in the peri- and postoperative course from peripheral blood was analyzed using a multiplex assay panel. The inflammatory marker IL-6 displays an increase in the OLR and RLR groups from T1 to T2, followed by a decrease in the postoperative course. There is no significant difference of IL-6 concentration between the RLR and OLR groups at any measured point of time, but a trend toward reduced cytokine levels at T2 in RLR (Fig. 3a). The concentration of IL-10 increases significantly in both groups between T1 and T2, followed by a significant decrease to the initial values at POD1. Patients undergoing RLR show lower levels of IL-10 at time point T2 (*g =* 0.68) compared to the OLR group (Fig. 3b). In both groups, there is no relevant alteration of CXCL10 levels, except a slight reduction of its systemic concentration in T1 and T2 in the RLR group (Fig. 3c). The concentration of MCP1 shows a trend for reduced chemokine levels in the RLR group with moderate effect size at T1 and T2 (*g =* 0.62) with a peak on POD1 (*g =* 0.54) and POD5 (*g =* 0.9) (Fig. 2d). IL-17a, IL-12p70, and IFNy do not display relevant differences in the cytokine levels in comparison between the groups or in the postoperative course (Fig. 3e-g).

In summary, IL-6, IL-10, and MCP1 show alterations in the peri- and postoperative cytokine levels based on the surgery itself. Reduced cytokine levels in the RLR group were observed for IL-6, IL-10, and CXCL10 in the perioperative course and MCP1 in the late postoperative course. To correlate cytokine response to local tissue damage, local liver and peritoneal cytokine levels were further analyzed.

### Cytokine and chemokine expression in the liver and peritoneal tissue

During surgery, liver and peritoneal biopsies were conducted to analyze the local inflammatory response of the tissue. T1 biopsy was collected prior to liver parenchyma dissection and T2 biopsy was obtained directly after the resection. The biopsies were homogenized and analyzed using a FACS-based multiplex assay panel for cytokines and chemokines.

**Fig. 4:**
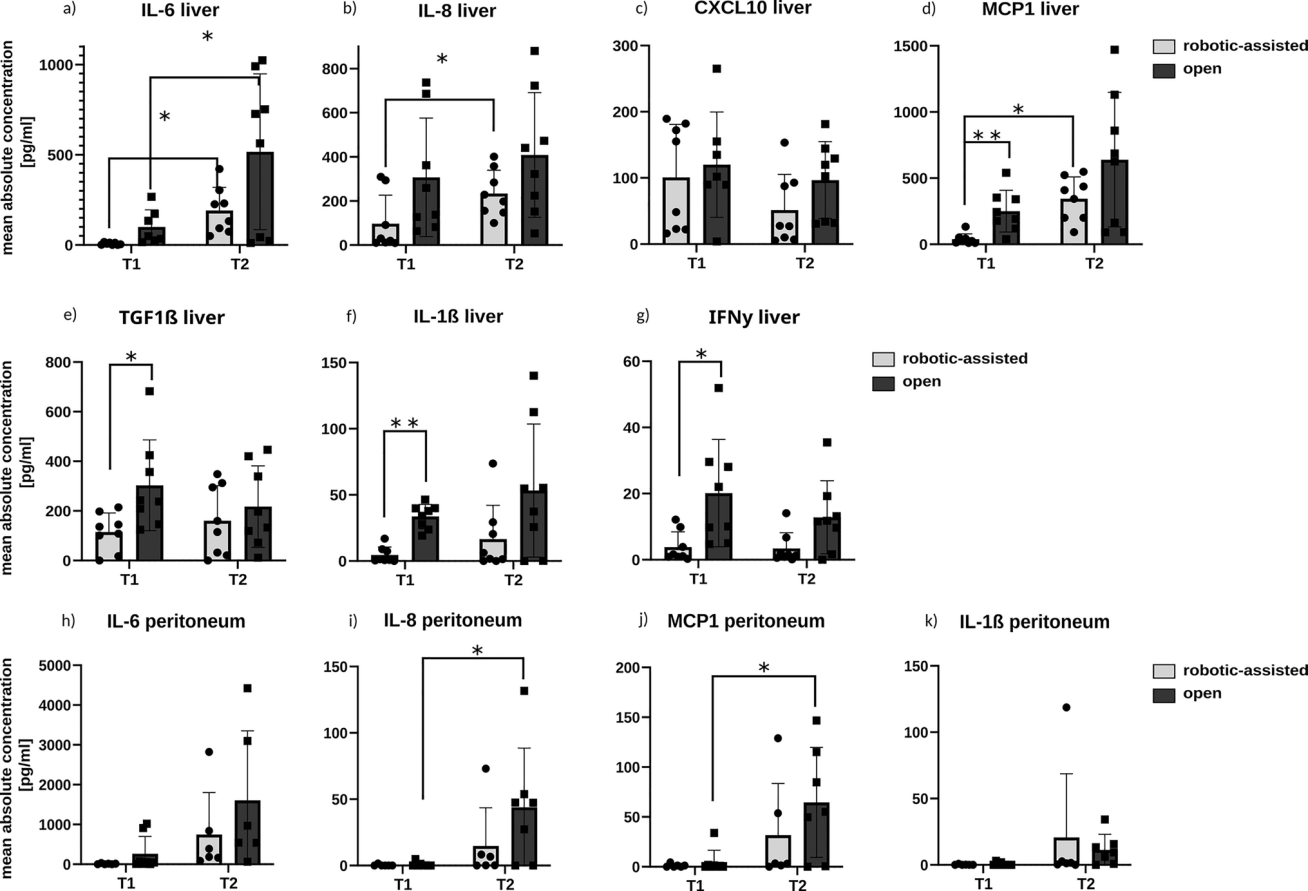
The concentration of selected regulatory proteins was measured in liver and peritoneal tissue on the initial phase of the surgery (T1) and at the end of surgery (T2). (**a**) The mean concentration of IL-6 in liver tissue; (**b**) the mean concentration of IL-8 in liver tissue; (**c**) the mean concentration of CXCL10 in liver tissue; (**d**) the mean concentration of MCP1 in liver tissue; (**e**) the mean concentration of TGF-1ß in liver tissue; (**f**) the mean concentration of IL-1ß in liver tissue; (**g**) the mean concentration of IFNy; (**h**) the mean concentration of IL-6 in peritoneal tissue; (**i**) the mean concentration of IL-8 in peritoneal tissue; (**j**) the mean concentration of MCP1 in peritoneal tissue; (**k**) the mean concentration of IL-1ß in peritoneal tissue. For an enhanced visual representation of our data, outliers, defined as extreme values differentiating fivefold of the interquartile range from the lower or upper quartile, have been excluded from the figures

In the initial phase of surgery, there is a relevant difference in cytokine and chemokine levels in the liver tissue. There are higher levels of IL-6 (*g =* 0.90), IL-8 (*g =* 1.00), and CXCL10 (*g =* 0.55) and a significantly higher level of IFNγ (*p =* 0.025, *g =* 1.37), TGFβ1 (*p =* 0.024, *g =* 1.34), MCP1 (*p =* 0.006, *g =* 1.84), and IL-1β (p ≤ 0.001, *g =* 3.72) in the OLR group, which is the result of the laparotomy and adhesiolysis (Fig. 4a-g). Furthermore, there is a significant increase of IL-6, IL-10, and MCP1 during surgery from T1 to T2. After liver parenchyma dissection, the cytokine and chemokine levels of IL-6 (*g =* 1.02), IL-8 (*g =* 0.82), IFNγ (*p =* 0.053, *g =* 1.11), MCP1 (*g =* 0.78), and IL-1β (*g =* 0.37) in the liver were higher in the OLR group. The levels of TGFβ1 (T1 *g =* 1.54) and CXCL10 (T1 *g =* 0.55, T2 *g =* 0.81) were also higher in the OLR group, whereas the dynamics of these cytokines and chemokines are stable or decreasing over the time of surgery, so that the levels mostly depend on the laparotomy stimulus. A supplementary subgroup analysis suggested a non-significant trend toward higher IL-6 levels at T1 (blood and liver tissue) in patients with prior liver resections with extensive adhesiolysis, which was not consistent at T2 or throughout other inflammatory parameters (Supplementary Table S2).

In peritoneal biopsies, a similar trend can be observed as described above. Prior to liver parenchyma dissection, there is a higher level of IL-6 (*g =* 0.77) in the OLR group and a similar trend at the end of surgery (*g =* 0.62) (Fig. 4h). An equivalent dynamic can be found for the concentration of IL-8 (T2 *g =* 0.82), MCP1 (T2 *g =* 0.65) and IL-1β in peritoneal tissue, displaying higher values at T2 (Fig. 4i-k).

## Discussion

This study provides a comparative analysis of systemic and local inflammatory responses in open and robotic-assisted liver surgery. Systemic inflammatory response was assessed via leucocyte populations showing reduced levels of CD4^+^/CD8^+^ lymphocytes in the RLR group in the late postoperative course. However, leucocyte stimulation efficacy, measured through L-selectin (CD62L) shedding, indicates a preserved immune competence in the RLR group in the postoperative course until POD20. Reduced systemic cytokine levels in the RLR group were observed for IL-6, IL-10, and CXCL10 in the perioperative course and MCP1 in the late postoperative course. Local inflammation was evaluated in liver and peritoneal biopsies taken before (T1) and after (T2) liver parenchyma dissection. Reduced inflammatory cytokines were observed in the RLR group, indicating less tissue trauma due to the trocar placement (T1: IL-6, IL-8, MCP1, IFNγ, TGF1β, IL-1β) and the surgical approach itself (T2: IL-6, IL-8, MCP1, IFNγ, IL-1β, and CXCL10). Peritoneal biopsies suggest similar trends in the perioperative course favoring the robotic approach (T2: IL-6, IL-8, MCP1). These results indicate reduced systemic inflammatory response, which may be a result of reduced tissue damage and consequently reduced cytokine release. While some differences were not statistically significant, the effect sizes (Hedge’s g) indicate substantial group differences, notably with regard to cytokine levels in liver tissue and CD62L expression after stimulation. This suggests a potential biological impact of the surgical approach, underlining the value of reporting effect sizes for complementary information alongside p-values.

Surgery triggers a complex inflammatory response crucial for wound healing. However, this response can also cause postoperative immunosuppression and possibly lead to tumor recurrence and metastasis in cases of long-term severe immunosuppression. Within hours, damage-associated molecular patterns trigger cytokine release (IL-6, IL-8, IL-10, and MCP1) while IFNγ levels decline [12]. Immunosuppression peaks in the first postoperative week and may last for months, particularly in cases of extensive surgical trauma, which is characterized by reduced NK cells and lymphocytes [12]. In our study, NK cell count was not altered, whereas reduced levels of CD4^+^/CD8^+^ lymphocytes were observed in the late postoperative course. The role of these lymphocytes is poorly understood. Some research indicates a protective role in tumor microenvironment, and others allocate an immunosuppressive effect leading to tumor progression [23, 24]. Differentiated CD4^+^/CD8^−^ and CD4^−^/CD8^+^ lymphocytes were not significantly different within the groups.

Functional immune assays, such as L-selectin shedding, further indicated preserved immune function in RLR, with a peak response at POD7. Increased L-selectin shedding is correlated with an effective immune response due to T-cell activation and their effective migration into inflamed tissue [25, 26]. In septic patients, high L-selectin shedding is crucial to limit local leukocyte adherence, microvascular leakage, and reduces the inflammatory response, leading to a reduced risk of end-organ injury due to sepsis [27]. Therefore, higher L-selectin shedding indicates preserved immune competence mitigating postoperative immunosuppression of patients in the RLR group, due to reduced activation of the immune system, especially in the late postoperative course.

In our cohort, robotic liver surgery took significantly longer than OLR, with the consequence of a slight increase in the need for mechanical ventilation and catecholamine support after surgery. However, RLR group patients displayed a reduced stay at the intensive care unit, which may be the result of a faster recovery due to the reduced inflammatory response to reduced surgical injury. This is further supported by reduced GLDH as a marker for hepatocyte injury in the RLR group in the early and late postoperative course. Previous research demonstrated lower systemic inflammation in MILS for HCC [14] and CRLM [17], though data for CCA remain scarce. Our findings substantiate reduced systemic IL-6 and MCP1 levels in RLR, similar to laparoscopic liver resections [14, 16, 17]. High IL-6 levels correlate with post-hepatectomy liver failure and prolonged recovery [28], seen in patients with extensive resections and complex adhesiolysis in our cohort. Higher MCP1 levels were detected in peritoneal biopsies in OLR, possibly due to surgical abrasion [29]. Lower IL-6 and IL-10 levels in RLR support its immunological benefits, as reported in colorectal surgery [30]. Cytokine profiling revealed higher CXCL10 levels in OLR liver tissue at T1, decreasing more in RLR, suggesting reduced inflammation and tissue damage. IL-10, as an anti-inflammatory and cytoprotective cytokine, was only detected in peripheral blood, where higher levels in OLR indicated increased systemic inflammation and, together with IL-6 and MCP1, suggested an increased risk of postoperative liver failure [28]. Similarly, IL-1β, associated with tumor-promoting inflammation, was elevated in OLR liver tissue but was absent in the blood [18].

IFNγ, which is associated with acute liver failure [31], exhibited higher levels in OLR at T1 in the liver, whereas RLR patients showed stable and slightly reduced postoperative levels in the blood, recovering by POD5. TGFβ1, a key inhibitor of hepatocyte proliferation [32], remained low and stable in RLR, whereas OLR samples showed an early increase with subsequent decline. These findings further support the immunological advantages of robotic-assisted surgery, which may also enhance liver regeneration.

This initial analysis of 11 patients per group showed clear trends in favor of RLR, although some results were not significant due to the variability of individual parameters. Consequently, the reported p-values should be interpreted with caution, as the relatively small cohort size may affect the statistical power of the findings. Furthermore, in the RLR group, many patients had prior radio- or chemotherapy, though only three had an interval of less than five months before surgery. Still, pre-existing immunosuppression from these treatments may have contributed to the observed differences in inflammatory response, potentially confounding the effect of the surgical technique. To overcome these limitations, future studies with larger patient cohorts are planned to validate the results. Another limitation is the limited insight into the postoperative local inflammatory response, as the tissue samples were only taken before and after resection. In comparison, the systemic inflammatory response could also be analyzed in the late postoperative course.

Surgical stress induced immunosuppression has an unfavorable oncological side effect, promoting cancer cell progression and awakening of dormant cancer cells [33]. High levels of inflammatory parameters like IL-6 are a risk factor for HCC recurrence [14]. Neutrophils, key responders to trauma, can contribute to tumor progression through extracellular trap formation [12]. While neutrophil counts were similar between RLR and OLR groups, lower inflammatory cytokine levels in RLR suggest reduced chemotaxis and their migration to the tissue trauma site. Excessive immune activation further recruits M2 macrophages, which enhance PD-L1-mediated immunosuppression via IL-10 and TGFβ1, thus reducing cytotoxic T-cell tumor infiltration and promoting tumor recurrence [34, 35]. Lower inflammatory responses with reduced MCP1, IL-6, and IL-10 levels in RLR may thus confer oncologic benefits, warranting further investigation. Strategies like perioperative immunotherapy [12] and enhanced recovery after surgery (ERAS) protocols [33] could further mitigate immunosuppression and improve long-term outcomes.

This study provides the first comparative analysis of systemic and local inflammatory responses in robotic-assisted liver surgery in the early and late perioperative course. Despite small sample sizes, clear trends suggest preserved immune competence and reduced cytokine release in RLR, particularly before and after liver resection. Future studies with larger cohorts and extended postoperative tissue analysis are needed to validate these findings and explore their oncological implications.

## Supplementary Information

Below is the link to the electronic supplementary material.

Supplementary file1 (DOCX 240 KB)

## Abbreviations

CCA: Cholangiocarcinoma
CD: Cluster of differentiation
CRLM: Colorectal liver metastases
CRP: C-reactive protein
CXCR: C-X-C chemokine receptor
FACS: Fluorescence activated cell sorting
GLDH: Glutamate dehydrogenase
HCC: Hepatocellular carcinoma
HLA-DR: Human leukocyte antigen- DR isotype
IFN: Interferon
IL: Interleukin
MCP: Monocyte chemoattractant protein
MILS: Minimally invasive liver surgery
OLR: Open liver resections
PMA: Phorbol 12-myristate 13-acetate
POD: Postoperative day
RLR: Robotic-assisted liver resections
RT: Room temperature
TGF: Transforming growth factor
TNF: Tumor necrosis factor
