# Supplementary material for: Robotic liver surgery: enhancing immune competence and minimizing postsurgical inflammation
Source: Surg Endosc. 2025 Sep 26;39(12):8336–48. doi: 10.1007/s00464-025-12195-1 (PMC12708820; doi:10.1007/s00464-025-12195-1)
Supplement: Supplementary file 1 — Supplementary file1 (DOCX 240 KB) [file 464_2025_12195_MOESM1_ESM.docx]

## **Robotic liver surgery: enhancing immune competence and minimizing postsurgical inflammation**

Julia Nagelschmitz^1^, Thomas Wartmann^1^, Severin Gylstorff^2^, Ahmed Sanin^1^, Ronny Otto^3^, Jörg Arend^1^, Mareike Franz^1^, Mirhasan Rahimli^1^, Andrew A. Gumbs^1,4^, Ulf D. Kahlert^1,5^, Frederike Stelter^1^ *, Roland S. Croner^1,4^ *

^1^ Clinic for General-, Visceral-, Vascular-, and Transplantation Surgery, Medical Faculty and University Medical Center Magdeburg, Leipziger Str. 44, D-39120 Magdeburg, Germany

^2^ Experimental Radiology, University Clinic for Radiology and Nuclear Medicine, Medical Faculty and University Medical Center Magdeburg, Leipziger Str. 44, D-39120 Magdeburg, Germany

^3^ Institute for Quality Assurance in Operative Medicine GmbH, Leipziger Str. 44, D-39120 Magdeburg, Germany

^4^ Service de Chirurgie Digestive Minimale Invasive, Hôpital Antoine Béclère, Assistance Publique-Hôpitaux de Paris, Clamart 92140 France

^5^ Research campus STIMULATE, Otto-von-Guericke University Magdeburg, Otto-Hahn-Straße 2, D-39106 Magdeburg, Germany

* These authors contributed equally

Correspondence: Frederike Stelter ([frederike.stelter@med.ovgu.de](mailto:frederike.stelter@med.ovgu.de))

ORCID-ID: 0000-0003-0110-4066

**Supplementary Materials**

| **Supplementary Methods** | *Page* |
| --- | --- |
| Multiplex analysis tool | *2* |
| **Supplementary Figures and Tables** |  |
| Table S1: Antibodies used in this study | *3* |
| Table S2: Inflammatory parameters in patients undergoing open liver resection with and without prior surgery of the liver |  |
| Table S3: Calculation of the effect size using Hedge’s g to consider non-symmetric sample size | *6* |
| Figure S1: Further laboratory parameter | *14* |

**Supplementary Methods**

**Multiplex analysis tool**

A multiplex assay panel was conducted on tissue homogenates and patient up to POD5 using the LEGENDplex HU Essential Immune Response Panel (13-plex) with a v-bottom plate for flow cytometry. After 1:2 dilution of the sample and assay buffer, 50 µl of the diluted sample was added to the v-bottom plate in duplicates and mixed with 50 µl of beads. The plate incubated for 2 hours at 800 rpm at RT, afterwards centrifuged at 250 xg for 5 min and washed with wash buffer. 25 µl of antibody mix was added and incubated for 1 hour at 800 rpm at RT. Subsequently, 25 µl of streptavidin phycoerythrin was transferred to each well and incubated for 30 min at 800 rpm at RT. The probes were centrifuged at 250 xg for 5 min and add 250 µl of wash buffer. The plate was read on a BD FACSCelesta (BD Biosciences) and analyte concentrations were evaluated

**Supplementary Figures and Tables**

**Table S1:** Antibodies used in this study

| Target | channel | Manufacturer / Catalog number | dilution | Mastermix 1: Lymphocytes | Mastermix 2:  Monocytes |
| --- | --- | --- | --- | --- | --- |
| CD3 | BV510 | BioLegend #317332 | 1: 75 | 1.5 µl | / |
| CD4 | PerCP/Cyanine5.5 | BioLegend #300530 | 1: 100 | 1.0 µl | / |
| CD8 | FITC | BioLegend #301006 | 1: 75 | 1.5 µl | / |
| CD56 | PE | BioLegend #362508 | 1: 75 | 1.5 µl | / |
| CD14 | PerCP/Cyanine5.5 | BioLegend #301824 | 1: 50 | / | 2.0 µl |
| CD16 | APC | BioLegend #302012 | 1: 75 | 1.5 µl | 1.5 µl |
| CD62L | APC/Cyanine7 | BioLegend #304814 | 1: 75 | 1.5 µl | 1.5 µl |
| HLA-DR | BV510 | BioLegend #307646 | 1: 50 | / | 2.0 µl |
| Zombie Violet | 423 nm | Biolegend #423113 | 1: 100 | / | / |

**Table S2:** Inflammatory parameters in patients undergoing open liver resection with and without a prior surgery of the liver.

|  | With previous liver surgery | Without previous liver surgery | all | p | n |
| --- | --- | --- | --- | --- | --- |
| sex |  |  |  | 0.567 | 11 |
| male | 2 (40.0) | 4 (66.7) | 6 (54.5) |  |  |
| female | 3 (60.0) | 2 (33.3) | 5 (45.5) |  |  |
| age at time of surgery [y] | 62.6±7.67 | 62.2±11.87 | 62.4±9.70 | 0.943 | 11 |
| BMI [kg/m^2] | 29.7±4.42 | 32.3±6.79 | 31.1±5.72 | 0.462 | 11 |
| neoadjuvant chemo-/radiotherapy | 2 (40.0) | 0 (0.00) | 2 (18.2) | 0.182 | 11 |
| surgery indication |  |  |  |  |  |
| primary tumor of the liver | 1 (20.0) | 4 (66.7) | 5 (45.5) | 0.242 | 11 |
| secondary tumor of the liver | 3 (60.0) | 1 (16.7) | 4 (36.4) | 0.242 | 11 |
| relapse | 3 (60.0) | 0 (7.69) | 3 (27.3) | 0.061 | 11 |
| cyst of the liver | 0 (0.00) | 1 (16.7) | 1 (9.09) | 1.000 | 11 |
| tumor of the gallblader | 1 (20.0) | 0 (0.00) | 1 (9.09) | 0.455 | 11 |
| ASA-score |  |  |  | 0.567 | 11 |
| 2 | 3 (60.0) | 2 (33.3) | 5 (45.5) |  |  |
| 3 | 2 (40.0) | 4 (66.7) | 6 (54.5) |  |  |
| duration of surgery | 221±74.0 | 221.5±72.5 | 221.3±69.4 | 0.991 | 11 |
| extent of surgery |  |  |  | 0.545 | 11 |
| major surgery | 1 (20.0) | 3 (50.0) | 4 (36.4) |  |  |
| minor surgery | 4 (80.0) | 3 (50.0) | 7 (63.6) |  |  |
| blood loss [ml] | 472±483 | 867±855 | 687±708 | 0.364 | 11 |
| time of pringle maneuver [min] | .±. | 33.0±. | 33.0±. | . | 1 |
| Clavien-Dindo-Score |  |  |  | 1.000 | 11 |
| < 3 | 3 (60.0) | 3 (50.0) | 6 (54.5) |  |  |
| ≥ 3 | 2 (40.0) | 3 (50.0) | 5 (45.5) |  |  |
| rehospitalisation within the first 30 days | 1 (20.0) | 1 (16.2) | 2 (18.2) | 1.000 | 11 |
| duration of stationary hospital stay [d] | 17.6±13.0 | 22.2±25.7 | 20.1±20.1 | 0.713 | 11 |
|  |  |  |  |  |  |
| IL-6 from blood at T1 [pg/ml] | 54.3±78.3 | 12.5±16.3 | 31.5±55.3 | 0.307 | 11 |
| IL-6 from blood at T2 [pg/ml] | 364±418 | 246±180 | 299±299 | 0.583 | 11 |
| IL-10 from blood at T1 [pg/ml] | 11.3±11.3 | 10.1±12.6 | 10.6±11.4 | 0.874 | 11 |
| IL-10 from blood at T2 [pg/ml] | 189±285 | 263±301 | 229±281 | 0.688 | 11 |
| IL-17a from blood at T1 [pg/ml] | 9.72±12.6 | 10.9±9.82 | 10.4±10.6 | 0.864 | 11 |
| IL-17a from blood at T2 [pg/ml] | 7.83±10.8 | 11.1±11.8 | 9.60±10.9 | 0.646 | 11 |
| IL-12p70 from blood at T1 [pg/ml] | 6.24±8.08 | 17.3±25.3 | 12.3±19.5 | 0.351 | 11 |
| IL-12p70 from blood at T2 [pg/ml] | 5.52±6.09 | 19.5±31.0 | 13.2±23.4 | 0.330 | 11 |
| CXCL-10 from blood at T1 [pg/ml] | 345±242 | 329±247 | 336±232 | 0.917 | 11 |
| CXCL-10 from blood at T2 [pg/ml] | 359±251 | 269±189 | 309±213 | 0.529 | 11 |
| MCP-1 from blood at T1 [pg/ml] | 473±436 | 340±292 | 401±351 | 0.578 | 11 |
| MCP-1 from blood at T2 [pg/ml] | 926±1280 | 1082±1541 | 1011±1360 | 0.859 | 11 |
| IFNy from blood at T1 [pg/ml] | 12.7±12.2 | 7.68±8.28 | 10.1±10.0 | 0.473 | 11 |
| IFNy from blood at T2 [pg/ml] | 11.7±15.9 | 4.19±3.42 | 7.59±11.0 | 0.359 | 11 |
|  |  |  |  |  |  |
| IL-6 in liver tissue at T1 [pg/ml] | 266±392 | 109±137 | 207±316 | 0.452 | 8 |
| IL-6 in liver tissue at T2 [pg/ml] | 418±538 | 681±102 | 517±432 | 0.349 | 8 |
| IL-8 in liver tissue at T1 [pg/ml] | 219±263 | 453±252 | 307±268 | 0.266 | 8 |
| IL-8 in liver tissue at T2 [pg/ml] | 383±255 | 452±381 | 408±283 | 0.799 | 8 |
| IFNy in liver tissue at T1 [pg/ml] | 13.9±10.6 | 30.5±20.9 | 20.1±16.2 | 0.291 | 8 |
| IFNy in liver tissue at T2 [pg/ml] | 11.1±6.36 | 15.7±18.1 | 12.8±11.0 | 0.714 | 8 |
| TGF-1ß in liver tissue at T1 [pg/ml] | 287±227 | 330±110 | 303±183 | 0.730 | 8 |
| TGF-1ß in liver tissue at T2 [pg/ml] | 238±153 | 184±212 | 217±164 | 0.726 | 8 |
| CXCL-10 in liver tissue at T1 [pg/ml] | 119±94.8 | 400±482 | 225±305 | 0.423 | 8 |
| CXCL-10 in liver tissue at T2 [pg/ml] | 113±54.4 | 70.0±64.4 | 96.7±58.0 | 0.389 | 8 |
| MCP-1 in liver tissue at T1 [pg/ml] | 260±192 | 235±111 | 251±158 | 0.819 | 8 |
| MCP-1 in liver tissue at T2 [pg/ml] | 471±357 | 922±677 | 640±508 | 0.364 | 8 |
| IL-1ß in liver tissue at T1 [pg/ml] | 35.5±10.2 | 30.5±8.28 | 33.6±9.24 | 0.481 | 8 |
| IL-1ß in liver tissue at T2 [pg/ml] | 63.2±60.0 | 36.7±31.8 | 53.3±50.3 | 0.466 | 8 |
| IL-6 in peritoneal tissue at T1 [pg/ml] | 228±443 | 329±503 | 266±433 | 0.788 | 8 |
| IL-6 in peritoneal tissue at T2 [pg/ml] | 1912±3109 | 15947±21117 | 7175±13627 | 0.371 | 8 |
| MCP-1 in peritoneal tissue at T1 [pg/ml] | 7.24±14.9 | 0.62±1.07 | 4.75±11.8 | 0.378 | 8 |
| MCP-1 in peritoneal tissue at T2 [pg/ml] | 157±245 | 83.9±54.5 | 130±191 | 0.548 | 8 |
| IL-1ß in peritoneal tissue at T1 [pg/ml] | 0.40±0.89 | 1.03±1.78 | 0.63±1.21 | 0.606 | 8 |
| IL-1ß in peritoneal tissue at T2 [pg/ml] | 11.6±13.9 | 167±271 | 69.9±166 | 0.425 | 8 |
| IL-8 in peritoneal tissue at T1 [pg/ml] | 1.01±2.18 | 0.45±0.79 | 0.80±1.72 | 0.626 | 8 |
| IL-8 in peritoneal tissue at T2 [pg/ml] | 52.0±49.2 | 102±137 | 70.7±86.3 | 0.606 | 8 |

**Table S3: Calculation of the effect size using Hedge’s g to consider non-symmetric sample size**

SD= Standard deviation, n= numbers of samples

Moderate effect ≤0.5/≥0.5 marked in yellow

Strong effect ≤0.8/≥0.8 marked in green

|  | **OLS** | | | **RLS** | | | **SD_pooled_** | **Hedge's g** |
| --- | --- | --- | --- | --- | --- | --- | --- | --- |
|  | **n** | **mean** | **SD** | **n** | **mean** | **SD** |  |  |
| BMI [kg/m^2] | 11 | 31,13 | 5,72 | 11 | 27,56 | 4,68 | 05,23 | 0,68 |
| blood loss [ml] | 11 | 687,27 | 707,80 | 11 | 658,18 | 525,60 | 623,39 | 0,05 |
| duration of intensive medical care [d] | 11 | 7,18 | 21,51 | 11 | 4,55 | 7,34 | 16,07 | 0,16 |
| duration of surgery [min] | 11 | 221,27 | 69,39 | 11 | 348,00 | 47,78 | 59,57 | -2,13 |
| duration of stationary hospital stay [d] | 11 | 20,09 | 20,08 | 11 | 16,09 | 9,97 | 15,85 | 0,25 |
|  |  |  |  |  |  |  |  |  |
| CRP T1 [mg/l] | 10 | 9,47 | 13,70 | 11 | 4,70 | 2,62 | 09,62 | 0,50 |
| CRP T2 [mg/l] | 6 | 5,79 | 6,18 | 8 | 4,69 | 2,63 | 04,47 | 0,25 |
| CRP on POD1 [mg/l] | 7 | 50,56 | 29,67 | 8 | 41,76 | 24,08 | 26,81 | 0,33 |
| CRP on average POD2 to POD7 [mg/l] | 11 | 104,18 | 68,50 | 11 | 142,00 | 80,52 | 74,75 | -0,51 |
| CRP on average one week after surgery [mg/l] | 9 | 126,40 | 115,59 | 8 | 93,45 | 39,02 | 88,52 | 0,37 |
| CRP after three weeks [mg/l] | 4 | 93,15 | 98,37 | 6 | 52,43 | 15,82 | 61,52 | 0,66 |
| Leukocytes T1 [Gpt/l] | 11 | 7,10 | 2,67 | 11 | 7,30 | 2,61 | 02,64 | -0,08 |
| LeukocytesT2 [Gpt/l] | 6 | 16,38 | 5,39 | 8 | 13,66 | 3,86 | 04,56 | 0,60 |
| Leukocytes on POD1 [Gpt/l] | 7 | 12,43 | 3,81 | 8 | 11,80 | 2,59 | 03,21 | 0,20 |
| Leukocytes on average POD2 to POD7 [Gpt/l] | 11 | 10,73 | 4,85 | 11 | 9,32 | 3,75 | 04,34 | 0,33 |
| Leukocytes on average one week after surgery [Gpt/l] | 9 | 11,59 | 6,42 | 8 | 14,11 | 7,26 | 06,82 | -0,37 |
| Leukocytes after three weeks [Gpt/l] | 5 | 8,61 | 4,87 | 6 | 10,00 | 6,57 | 05,88 | -0,24 |
| ALAT T1 [µmol/l*s] | 11 | ,46 | ,39 | 11 | ,40 | ,15 | 00,30 | 0,20 |
| ALAT T2 [µmol/l*s] | 6 | 7,05 | 3,08 | 8 | 6,81 | 6,36 | 05,25 | 0,05 |
| ALAT on POD1 [µmol/l*s] | 7 | 13,75 | 10,11 | 8 | 10,01 | 7,86 | 08,97 | 0,42 |
| ALAT on average POD2 to POD7 [µmol/l*s] | 11 | 5,41 | 5,31 | 11 | 4,24 | 2,41 | 04,12 | 0,28 |
| ALAT on average one week after surgery [µmol/l*s] | 9 | 2,08 | 2,33 | 8 | 1,30 | ,86 | 01,80 | 0,43 |
| ALAT after three weeks [µmol/l*s] | 5 | 4,47 | 8,69 | 4 | ,83 | ,58 | 06,58 | 0,55 |
| ASAT T1 [µmol/l*s] | 11 | ,50 | ,24 | 11 | ,61 | ,40 | 00,33 | -0,33 |
| ASAT T2 [µmol/l*s] | 5 | 10,08 | 1,87 | 3 | 9,23 | 7,17 | 04,41 | 0,19 |
| ASAT on POD1 [µmol/l*s] | 5 | 17,03 | 12,53 | 6 | 12,38 | 5,88 | 09,43 | 0,49 |
| ASAT on average POD2 to POD7 [µmol/l*s] | 11 | 3,47 | 3,67 | 11 | 3,23 | 3,40 | 03,54 | 0,07 |
| ASAT on average one week after surgery [µmol/l*s] | 9 | 3,39 | 6,07 | 7 | 1,19 | 1,09 | 04,64 | 0,47 |
| ASAT after three weeks [µmol/l*s] | 3 | ,58 | ,20 | 4 | 1,43 | 1,36 | 01,06 | -0,80 |
| GLDH T1 [nmol/l*s] | 8 | 159,55 | 231,38 | 7 | 72,54 | 67,05 | 175,79 | 0,49 |
| GLDH T2 [nmol/l*s] | 5 | 2903,00 | 1001,52 | 3 | 778,67 | 331,43 | 839,83 | 2,53 |
| GLDH on POD1 [nmol/l*s] | 5 | 7584,40 | 7415,59 | 6 | 4046,67 | 1807,37 | 5123,98 | 0,69 |
| GLDH on average POD2 to POD7 [nmol/l*s] | 11 | 2437,58 | 2567,21 | 11 | 2077,63 | 1775,57 | 2207,17 | 0,16 |
| GLDH on average one week after surgery [nmol/l*s] | 9 | 1000,67 | 1748,04 | 5 | 310,09 | 342,22 | 1440,88 | 0,48 |
| GLDH after three weeks [nmol/l*s] | 2 | 110,78 | 12,41 | 4 | 99,86 | 52,60 | 45,97 | 0,24 |
| Bilirubin T1 [µmol/l] | 11 | 9,41 | 7,08 | 11 | 9,12 | 2,67 | 05,35 | 0,05 |
| Bilirubin T2 [µmol/l] | 6 | 26,15 | 3,08 | 8 | 21,78 | 9,45 | 07,49 | 0,58 |
| Bilirubin on POD1 [µmol/l] | 7 | 14,03 | 5,30 | 8 | 18,55 | 12,99 | 10,19 | -0,44 |
| Bilirubin on average POD2 to POD7 [µmol/l] | 11 | 8,55 | 3,69 | 11 | 25,38 | 33,85 | 24,08 | -0,70 |
| Bilirubin on average one week after surgery [µmol/l] | 9 | 8,18 | 5,65 | 8 | 38,88 | 64,97 | 44,57 | -0,69 |
| Bilirubin after three weeks [µmol/l] | 4 | 27,00 | 41,44 | 5 | 77,16 | 149,92 | 116,53 | -0,43 |
| gGT T1 [µmol/l*s] | 2 | 1,04 | ,47 | 4 | 1,36 | 1,65 | 01,45 | -0,22 |
| gGT T2 [µmol/l*s] | 5 | 2,35 | 2,53 | 3 | 1,73 | 1,04 | 02,15 | 0,29 |
| gGT on POD1 [µmol/l*s] | 5 | 2,29 | 2,16 | 6 | 1,29 | ,85 | 01,57 | 0,64 |
| gGT on average POD2 to POD7 [µmol/l*s] | 11 | 2,25 | 1,30 | 11 | 2,15 | 1,41 | 01,36 | 0,07 |
| gGT on average one week after surgery [µmol/l*s] | 9 | 3,09 | 1,31 | 6 | 4,67 | 4,56 | 03,01 | -0,53 |
| gGT after three weeks [µmol/l*s] | 3 | 5,48 | 6,60 | 5 | 4,29 | 4,22 | 05,14 | 0,23 |
|  |  |  |  |  |  |  |  |  |
| CD14+/CD16- cells from blood at T1 [%] | 11 | 60,35 | 12,41 | 11 | 66,55 | 10,87 | 11,67 | -0,53 |
| CD14+/CD16- cells from blood at T2 [%] | 11 | 51,01 | 17,15 | 11 | 67,84 | 16,13 | 16,65 | -1,01 |
| CD14+/CD16- cells from blood on POD1 [%] | 11 | 67,44 | 11,88 | 10 | 74,90 | 9,87 | 10,97 | -0,68 |
| CD14+/CD16- cells from blood on POD3 [%] | 11 | 69,99 | 15,44 | 9 | 79,24 | 7,57 | 12,57 | -0,74 |
| CD14+/CD16- cells from blood on POD5 [%] | 11 | 70,41 | 11,78 | 7 | 79,00 | 7,34 | 10,34 | -0,83 |
| CD14+/CD16- cells from blood on POD7 [%] | 8 | 66,14 | 19,61 | 7 | 69,40 | 13,25 | 16,97 | -0,19 |
| CD14+/CD16- cells from blood on POD20 [%] | 5 | 75,38 | 11,15 | 3 | 58,60 | 14,17 | 12,24 | 1,37 |
| CD14-/CD16+ cells from blood at T1 [%] | 11 | 15,86 | 12,94 | 11 | 16,35 | 10,41 | 11,74 | -0,04 |
| CD14-/CD16+ cells from blood at T2 [%] | 11 | 27,98 | 21,85 | 11 | 19,55 | 16,04 | 19,17 | 0,44 |
| CD14-/CD16+ cells from blood on POD1 [%] | 11 | 14,90 | 10,72 | 10 | 8,12 | 8,36 | 09,67 | 0,70 |
| CD14-/CD16+ cells from blood on POD3 [%] | 11 | 12,17 | 16,45 | 9 | 7,78 | 4,62 | 12,64 | 0,35 |
| CD14-/CD16+ cells from blood on POD5 [%] | 11 | 9,83 | 11,03 | 7 | 9,10 | 5,45 | 09,34 | 0,08 |
| CD14-/CD16+ cells from blood on POD7 [%] | 8 | 10,10 | 9,86 | 7 | 10,24 | 6,51 | 08,48 | -0,02 |
| CD14-/CD16+ cells from blood on POD20 [%] | 5 | 8,30 | 3,92 | 3 | 18,03 | 6,39 | 04,88 | -1,99 |
| CD14+/CD16+ cells from blood at T1 [%] | 11 | 2,19 | 1,48 | 11 | 4,40 | 2,70 | 02,18 | -1,02 |
| CD14+/CD16+ cells from blood at T2 [%] | 11 | 1,11 | 1,00 | 11 | 1,94 | 1,44 | 01,24 | -0,67 |
| CD14+/CD16+ cells from blood on POD1 [%] | 11 | 5,49 | 3,31 | 10 | 8,32 | 4,30 | 03,81 | -0,74 |
| CD14+/CD16+ cells from blood on POD3 [%] | 11 | 4,44 | 2,74 | 9 | 6,99 | 4,75 | 03,77 | -0,68 |
| CD14+/CD16+ cells from blood on POD5 [%] | 11 | 5,21 | 4,23 | 7 | 5,40 | 4,05 | 04,16 | -0,05 |
| CD14+/CD16+ cells from blood on POD7 [%] | 8 | 4,19 | 2,32 | 7 | 4,96 | 2,97 | 02,64 | -0,29 |
| CD14+/CD16+ cells from blood on POD20 [%] | 5 | 3,34 | 1,72 | 3 | 7,07 | 2,15 | 01,87 | -1,99 |
| CD14-/CD16- cells from blood at T1 [%] | 11 | 21,61 | 8,75 | 11 | 12,70 | 7,54 | 01,87 | -1,99 |
| CD14-/CD16- cells from blood at T2 [%] | 11 | 19,89 | 10,51 | 11 | 10,64 | 9,41 | 08,17 | 1,09 |
| CD14-/CD16- cells from blood on POD1 [%] | 11 | 11,45 | 6,43 | 10 | 8,66 | 6,26 | 09,98 | 0,93 |
| CD14-/CD16- cells from blood on POD3 [%] | 11 | 13,40 | 9,12 | 9 | 6,00 | 2,87 | 06,35 | 0,44 |
| CD14-/CD16- cells from blood on POD5 [%] | 11 | 14,55 | 7,42 | 7 | 6,47 | 3,82 | 07,06 | 1,05 |
| CD14-/CD16- cells from blood on POD7 [%] | 8 | 19,56 | 11,81 | 7 | 15,39 | 10,37 | 06,32 | 1,28 |
| CD14-/CD16- cells from blood on POD20 [%] | 5 | 13,02 | 8,17 | 3 | 16,33 | 7,39 | 11,17 | 0,37 |
| HLA-DR+ cells from blood at T1 [%] | 11 | 19,83 | 8,59 | 11 | 19,57 | 7,32 | 07,92 | -0,42 |
| HLA-DR+ cells from blood at T2 [%] | 11 | 14,47 | 7,26 | 11 | 18,06 | 8,36 | 07,83 | -0,46 |
| HLA-DR+ cells from blood on POD1 [%] | 11 | 26,05 | 8,70 | 10 | 26,43 | 11,00 | 09,86 | -0,04 |
| HLA-DR+ cells from blood on POD3 [%] | 11 | 27,49 | 9,78 | 9 | 27,62 | 10,21 | 09,97 | -0,01 |
| HLA-DR+ cells from blood on POD5 [%] | 11 | 25,43 | 8,73 | 7 | 30,06 | 4,34 | 07,40 | -0,63 |
| HLA-DR+ cells from blood on POD7 [%] | 8 | 25,40 | 9,41 | 7 | 25,90 | 7,60 | 08,62 | -0,06 |
| HLA-DR+ cells from blood on POD20 [%] | 5 | 23,92 | 5,81 | 3 | 24,13 | 8,03 | 06,63 | -0,03 |
| CD4+/CD8- cells from blood at T1 [%] | 11 | 59,11 | 11,05 | 11 | 49,76 | 12,24 | 11,66 | 0,80 |
| CD4+/CD8- cells from blood at T2 [%] | 11 | 49,62 | 12,85 | 11 | 52,86 | 13,36 | 13,11 | -0,25 |
| CD4+/CD8- cells from blood on POD1 [%] | 11 | 56,00 | 12,57 | 10 | 56,65 | 10,63 | 11,69 | -0,06 |
| CD4+/CD8- cells from blood on POD3 [%] | 11 | 60,74 | 13,27 | 9 | 63,23 | 14,04 | 13,62 | -0,18 |
| CD4+/CD8- cells from blood on POD5 [%] | 11 | 60,52 | 14,52 | 7 | 66,70 | 10,48 | 13,15 | -0,47 |
| CD4+/CD8- cells from blood on POD7 [%] | 8 | 62,45 | 8,82 | 7 | 64,19 | 10,78 | 09,77 | -0,18 |
| CD4+/CD8- cells from blood on POD20 [%] | 5 | 58,76 | 11,52 | 3 | 64,20 | 12,74 | 11,94 | -0,46 |
| CD4-/CD8+ cells from blood at T1 [%] | 11 | 28,13 | 11,53 | 11 | 37,41 | 13,75 | 12,69 | -0,73 |
| CD4-/CD8+ cells from blood at T2 [%] | 11 | 33,65 | 13,56 | 11 | 35,60 | 14,25 | 13,91 | -0,14 |
| CD4-/CD8+ cells from blood on POD1 [%] | 11 | 32,37 | 14,26 | 10 | 31,70 | 10,80 | 12,74 | 0,05 |
| CD4-/CD8+ cells from blood on POD3 [%] | 11 | 26,39 | 11,40 | 9 | 26,64 | 13,30 | 12,28 | -0,02 |
| CD4-/CD8+ cells from blood on POD5 [%] | 11 | 26,65 | 13,60 | 7 | 24,87 | 9,14 | 12,12 | 0,15 |
| CD4-/CD8+ cells from blood on POD7 [%] | 8 | 25,76 | 9,81 | 7 | 24,01 | 10,50 | 10,13 | 0,17 |
| CD4-/CD8+ cells from blood on POD20 [%] | 5 | 30,86 | 13,72 | 3 | 26,47 | 9,59 | 12,50 | 0,35 |
| CD4+/CD8+ cells from blood at T1 [%] | 11 | 6,51 | 3,41 | 11 | 4,74 | 3,36 | 03,39 | 0,52 |
| CD4+/CD8+ cells from blood at T2 [%] | 11 | 7,55 | 4,80 | 11 | 4,65 | 2,07 | 03,70 | 0,78 |
| CD4+/CD8+ cells from blood on POD1 [%] | 11 | 5,24 | 2,42 | 10 | 4,30 | 2,53 | 02,47 | 0,38 |
| CD4+/CD8+ cells from blood on POD3 [%] | 11 | 7,08 | 5,40 | 9 | 4,77 | 2,83 | 04,45 | 0,52 |
| CD4+/CD8+ cells from blood on POD5 [%] | 11 | 6,75 | 3,78 | 7 | 3,20 | ,73 | 03,02 | 1,17 |
| CD4+/CD8+ cells from blood on POD7 [%] | 8 | 6,42 | 4,48 | 7 | 6,06 | 2,77 | 03,79 | 0,10 |
| CD4+/CD8+ cells from blood on POD20 [%] | 5 | 6,20 | 3,79 | 3 | 3,43 | 1,97 | 03,30 | 0,84 |
| CD4-/CD8- cells from blood at T1 [%] | 11 | 6,25 | 3,59 | 11 | 8,07 | 6,34 | 05,15 | -0,35 |
| CD4-/CD8- cells from blood at T2 [%] | 11 | 9,16 | 6,68 | 11 | 6,91 | 4,83 | 05,83 | 0,39 |
| CD4-/CD8- cells from blood on POD1 [%] | 11 | 6,36 | 4,87 | 10 | 7,36 | 5,83 | 05,35 | -0,19 |
| CD4-/CD8- cells from blood on POD3 [%] | 11 | 5,80 | 3,76 | 9 | 5,33 | 3,49 | 03,64 | 0,13 |
| CD4-/CD8- cells from blood on POD5 [%] | 11 | 6,05 | 5,64 | 7 | 5,23 | 3,35 | 04,91 | 0,17 |
| CD4-/CD8- cells from blood on POD7 [%] | 8 | 5,46 | 3,90 | 7 | 5,77 | 4,55 | 04,21 | -0,07 |
| CD4-/CD8- cells from blood on POD20 [%] | 5 | 4,20 | 1,74 | 3 | 5,90 | 4,05 | 02,74 | -0,62 |
| CD56+/CD3- cells from blood at T1 [%] | 11 | 15,64 | 7,23 | 11 | 18,30 | 7,89 | 07,57 | -0,35 |
| CD56+/CD3- cells from blood at T2 [%] | 11 | 23,84 | 14,04 | 11 | 24,21 | 13,62 | 13,83 | -0,03 |
| CD56+/CD3- cells from blood on POD1 [%] | 11 | 9,34 | 4,19 | 10 | 10,87 | 7,12 | 05,77 | -0,27 |
| CD56+/CD3- cells from blood on POD3 [%] | 11 | 7,47 | 3,20 | 9 | 8,72 | 5,67 | 04,47 | -0,28 |
| CD56+/CD3- cells from blood on POD5 [%] | 11 | 9,47 | 5,62 | 7 | 9,57 | 6,78 | 06,08 | -0,02 |
| CD56+/CD3- cells from blood on POD7 [%] | 8 | 8,29 | 3,32 | 7 | 12,49 | 10,23 | 07,36 | -0,57 |
| CD56+/CD3- cells from blood on POD20 [%] | 5 | 10,60 | 7,00 | 3 | 8,17 | 3,15 | 06,00 | 0,41 |
| difference of CD62L+ cells from blood at T1 [%] | 11 | 8,91 | 6,42 | 11 | 11,98 | 6,12 | 06,27 | -0,49 |
| difference of CD62L+ cells from blood at T2 [%] | 11 | 7,14 | 5,04 | 11 | 10,09 | 6,81 | 05,99 | -0,49 |
| difference of CD62L+ cells from blood on POD1 [%] | 11 | 11,96 | 5,20 | 10 | 14,93 | 7,99 | 06,67 | -0,45 |
| difference of CD62L+ cells from blood on POD3 [%] | 11 | 15,47 | 6,55 | 9 | 15,79 | 9,79 | 08,15 | -0,04 |
| difference of CD62L+ cells from blood on POD5 [%] | 11 | 8,91 | 8,06 | 7 | 17,70 | 10,67 | 09,13 | -0,96 |
| difference of CD62L+ cells from blood on POD7 [%] | 8 | 10,50 | 9,43 | 7 | 19,41 | 10,34 | 09,86 | -0,90 |
| difference of CD62L+ cells from blood on POD20 [%] | 5 | 11,14 | 8,39 | 3 | 17,50 | 7,01 | 07,96 | -0,80 |
|  |  |  |  |  |  |  |  |  |
| IL-6 from blood at T1 [pg/ml] | 11 | 31,48 | 55,33 | 11 | 10,37 | 8,84 | 39,62 | 0,53 |
| IL-6 from blood at T2 [pg/ml] | 11 | 299,43 | 299,33 | 11 | 204,94 | 287,67 | 293,56 | 0,32 |
| IL-6 from blood on POD1 [pg/ml] | 11 | 247,26 | 216,10 | 10 | 542,31 | 1042,64 | 734,52 | -0,40 |
| IL-6 from blood on POD3 [pg/ml] | 11 | 584,94 | 1338,97 | 9 | 353,33 | 552,42 | 1063,79 | 0,22 |
| IL-6 from blood on POD5 [pg/ml] | 10 | 248,52 | 345,94 | 7 | 2267,17 | 5270,83 | 3344,32 | -0,60 |
| IL-10 from blood at T1 [pg/ml] | 11 | 10,64 | 11,43 | 11 | 9,26 | 8,36 | 10,01 | 0,14 |
| IL-10 from blood at T2 [pg/ml] | 11 | 229,19 | 281,42 | 11 | 90,36 | 73,31 | 205,64 | 0,68 |
| IL-10 from blood on POD1 [pg/ml] | 11 | 18,12 | 19,26 | 10 | 13,41 | 10,20 | 15,64 | 0,30 |
| IL-10 from blood on POD3 [pg/ml] | 11 | 12,61 | 13,34 | 9 | 7,39 | 7,40 | 11,10 | 0,47 |
| IL-10 from blood on POD5 [pg/ml] | 11 | 23,05 | 45,63 | 7 | 16,55 | 25,51 | 39,31 | 0,17 |
| IL-17a from blood at T1 [pg/ml] | 11 | 10,39 | 10,59 | 11 | 10,80 | 13,38 | 12,07 | -0,03 |
| IL-17a from blood at T2 [pg/ml] | 11 | 9,60 | 10,90 | 11 | 10,72 | 16,80 | 14,16 | -0,08 |
| IL-17a from blood on POD1 [pg/ml] | 11 | 8,37 | 9,49 | 10 | 10,83 | 14,67 | 12,22 | -0,20 |
| IL-17a from blood on POD3 [pg/ml] | 11 | 9,61 | 9,05 | 9 | 12,15 | 15,91 | 12,57 | -0,20 |
| IL-17a from blood on POD5 [pg/ml] | 11 | 17,36 | 27,61 | 7 | 18,69 | 21,36 | 25,45 | -0,05 |
| IL-12p70 from blood at T1 [pg/ml] | 11 | 12,27 | 19,47 | 11 | 9,60 | 8,84 | 15,12 | 0,18 |
| IL-12p70 from blood at T2 [pg/ml] | 11 | 13,15 | 23,44 | 11 | 10,50 | 12,40 | 18,75 | 0,14 |
| IL-12p70 from blood on POD1 [pg/ml] | 11 | 12,27 | 21,88 | 10 | 11,15 | 12,36 | 18,01 | 0,06 |
| IL-12p70 from blood on POD3 [pg/ml] | 11 | 14,05 | 25,14 | 9 | 8,90 | 9,75 | 19,83 | 0,26 |
| IL-12p70 from blood on POD5 [pg/ml] | 11 | 33,92 | 93,76 | 7 | 12,74 | 11,85 | 74,48 | 0,28 |
| CXCL-10 from blood at T1 [pg/ml] | 11 | 335,83 | 232,19 | 11 | 344,69 | 328,20 | 284,28 | -0,03 |
| CXCL-10 from blood at T2 [pg/ml] | 11 | 309,38 | 212,51 | 11 | 230,72 | 102,89 | 166,95 | 0,47 |
| CXCL-10 from blood on POD1 [pg/ml] | 11 | 230,64 | 149,37 | 10 | 243,74 | 220,89 | 186,70 | -0,07 |
| CXCL-10 from blood on POD3 [pg/ml] | 11 | 335,94 | 210,73 | 9 | 313,95 | 225,39 | 217,37 | 0,10 |
| CXCL-10 from blood on POD5 [pg/ml] | 11 | 403,11 | 332,27 | 7 | 341,08 | 227,97 | 297,47 | 0,21 |
| MCP-1 from blood at T1 [pg/ml] | 11 | 400,48 | 351,41 | 11 | 230,68 | 165,14 | 274,55 | 0,62 |
| MCP-1 from blood at T2 [pg/ml] | 11 | 1011,41 | 1359,88 | 11 | 387,23 | 387,68 | 999,89 | 0,62 |
| MCP-1 from blood on POD1 [pg/ml] | 11 | 484.33 | 465 | 9 | 283.46 | 196.43 | 370.5 | 0.54 |
| MCP-1 from blood on POD3 [pg/ml] | 11 | 297,88 | 316,69 | 9 | 231,42 | 105,56 | 246,31 | 0,27 |
| MCP-1 from blood on POD5 [pg/ml] | 11 | 515.56 | 500.54 | 6 | 147.37 | 55.92 | 409.96 | 0.90 |
| IFNy from blood at T1 [pg/ml] | 11 | 10,07 | 10,01 | 11 | 6,82 | 6,50 | 08,44 | 0,39 |
| IFNy from blood at T2 [pg/ml] | 11 | 7,59 | 11,04 | 11 | 6,80 | 7,96 | 09,62 | 0,08 |
| IFNy from blood on POD1 [pg/ml] | 11 | 6,71 | 5,94 | 10 | 7,61 | 8,57 | 07,30 | -0,12 |
| IFNy from blood on POD3 [pg/ml] | 11 | 7,34 | 6,33 | 9 | 4,66 | 4,71 | 05,67 | 0,47 |
| IFNy from blood on POD5 [pg/ml] | 11 | 7,80 | 6,88 | 7 | 6,76 | 5,78 | 06,49 | 0,16 |
| IL-6 in liver tissue at T1 [pg/ml] | 8 | 207,27 | 315,70 | 8 | 7,04 | 6,45 | 223,28 | 0,90 |
| IL-6 in liver tissue at T2 [pg/ml] | 8 | 516,74 | 431,96 | 8 | 191,36 | 128,43 | 318,66 | 1,02 |
| IL-8 in liver tissue at T1 [pg/ml] | 8 | 306,77 | 268,50 | 8 | 96,90 | 129,07 | 210,66 | 1,00 |
| IL-8 in liver tissue at T2 [pg/ml] | 8 | 408,49 | 282,90 | 8 | 233,79 | 105,73 | 213,55 | 0,82 |
| IFNy in liver tissue at T1 [pg/ml] | 8 | 20,15 | 16,22 | 8 | 3,82 | 4,60 | 11,92 | 1,37 |
| IFNy in liver tissue at T2 [pg/ml] | 8 | 12,82 | 11,05 | 8 | 3,40 | 4,78 | 08,51 | 1,11 |
| TGF-1ß in liver tissue at T1 [pg/ml] | 8 | 303,13 | 183,01 | 8 | 115,06 | 76,62 | 140,29 | 1,34 |
| TGF-1ß in liver tissue at T2 [pg/ml] | 8 | 217,38 | 164,28 | 8 | 160,41 | 141,50 | 153,31 | 0,37 |
| CXCL-10 in liver tissue at T1 [pg/ml] | 8 | 224,53 | 304,56 | 8 | 100,99 | 79,85 | 222,64 | 0,55 |
| CXCL-10 in liver tissue at T2 [pg/ml] | 8 | 96,74 | 58,02 | 8 | 51,36 | 53,97 | 56,03 | 0,81 |
| MCP-1 in liver tissue at T1 [pg/ml] | 8 | 250,75 | 157,68 | 8 | 39,16 | 40,77 | 115,16 | 1,84 |
| MCP-1 in liver tissue at T2 [pg/ml] | 8 | 639,94 | 508,38 | 8 | 344,27 | 165,47 | 378,04 | 0,78 |
| IL-1ß in liver tissue at T1 [pg/ml] | 8 | 33,63 | 9,24 | 8 | 4,55 | 6,07 | 07,82 | 3,72 |
| IL-1ß in liver tissue at T2 [pg/ml] | 8 | 53,26 | 50,31 | 8 | 16,57 | 25,48 | 39,88 | 0,92 |
| IL-6 in peritoneal tissue at T1 [pg/ml] | 8 | 265,86 | 432,51 | 6 | 10,36 | 11,11 | 330,41 | 0,77 |
| IL-6 in peritoneal tissue at T2 [pg/ml] | 8 | 7175,04 | 13626,70 | 6 | 748,44 | 1052,66 | 10429,72 | 0,62 |
| MCP-1 in peritoneal tissue at T1 [pg/ml] | 8 | 4,75 | 11,78 | 6 | 1,21 | 1,70 | 09,06 | 0,39 |
| MCP-1 in peritoneal tissue at T2 [pg/ml] | 8 | 129,82 | 191,48 | 6 | 31,73 | 51,90 | 150,03 | 0,65 |
| IL-1ß in peritoneal tissue at T1 [pg/ml] | 8 | ,63 | 1,21 | 6 | ,17 | ,31 | 00,95 | 0,49 |
| IL-1ß in peritoneal tissue at T2 [pg/ml] | 8 | 69,88 | 165,84 | 6 | 20,74 | 48,00 | 130,40 | 0,38 |
| IL-8 in peritoneal tissue at T1 [pg/ml] | 8 | ,80 | 1,72 | 6 | ,29 | ,64 | 01,38 | 0,37 |
| IL-8 in peritoneal tissue at T2 [pg/ml] | 8 | 70,70 | 86,28 | 6 | 14,75 | 28,80 | 68,47 | 0,82 |


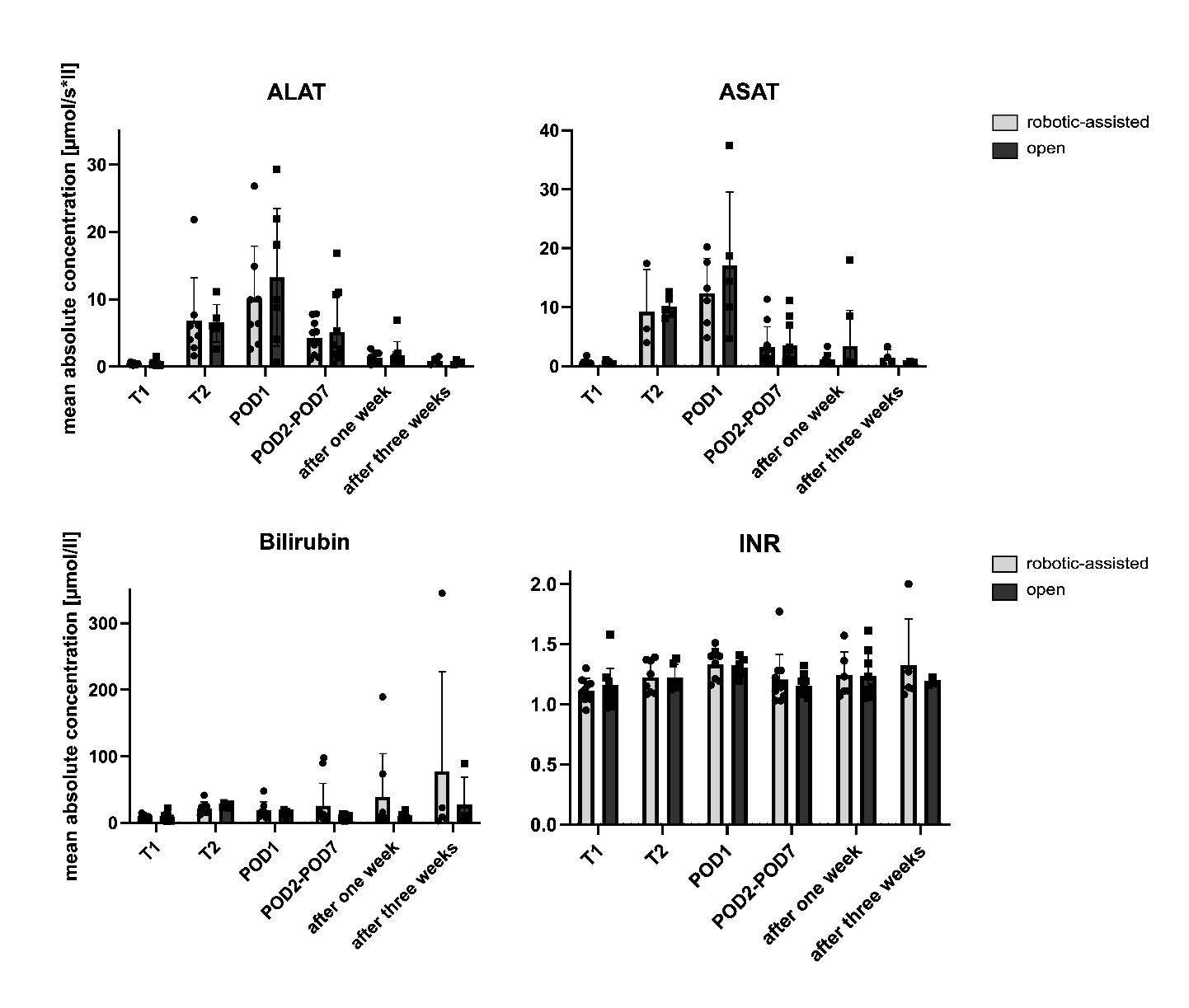


**Figure S1: Further laboratory parameters in both patient cohorts in the peri- and late postoperative course.** Mean absolute concentrations of ALAT, ASAT, bilirubin, and INR of patients in the peri- and late postoperative course.
